# A SYSTEMATIC REVIEW OF ENABLERS AND BARRIERS TO CANCER SURVIVORSHIP IN NIGERIA

**DOI:** 10.1093/geroni/igad104.1933

**Published:** 2023-12-21

**Authors:** Candidus Nwakasi, Kafayat Mahmoud, Darlingtina Esiaka

**Affiliations:** University of Connecticut, Storrs, Connecticut, United States; Boston University, Boston, Massachusetts, United States; University of Kentucky, Lexington, Kentucky, United States

## Abstract

**Background:**

Nigeria is benefitting from public health and healthcare advancement which is increasing cancer care in the country. As a result, more people are surviving cancer. However, this should not mask the fact that Nigeria, a rapidly aging country which an increased risk of cancer incidence, bears a heavy health burden of about 72,000 cancer deaths per year — this underscores cancer as a leading cause of death among Nigerians. The purpose of this study was to identify and synthesize factors that are enablers or barriers to cancer survivorship in Nigeria and add to the body of knowledge on cancer survivorship in the global south. Method. Using PRISMA guidelines, we conducted a systematic review across Scopus, PubMed, and databases. Given that cancer survivorship covers periods from diagnoses, to treatment, and end of life, we identified 31 peer-reviewed studies that assessed cancer treatment, management, care, and survivorship in Nigeria.

**Results:**

Eight themes emerged from our review of 31 peer-reviewed studies that examined factors that are enablers of barriers to cancer survivorship among Nigerians. Some of the themes include a desire to survive, the availability of treatment options, self-management, and the availability of pseudo-healthcare providers. We later grouped the themes under three categories, healthcare, economic, and psychological factors.

**Conclusion:**

Cancer survivors in Nigeria are confronted with several issues that affect their quality of life and health outcomes. This highlights the need for studies on cancer survivorship to inform the design of tailored health improvement interventions for cancer survivors in Nigeria.

